# Giant pleomorphic lipoma in patient with multiple osteochondromatosis

**DOI:** 10.1093/jscr/rjae353

**Published:** 2024-05-30

**Authors:** Benjamin Thorpe, Paloma Lage, Carolina Beiras, Kelly Bargas-Osorio, Francisco Canseco

**Affiliations:** General Surgery Department, Hospital Clinico Universitario de Santiago de Compostela, Rua Choupana s/n, Santiago de Compostela 15706, Spain; Plastic Surgery Department, Hospital Clinico Universitario de Santiago de Compostela, Rua Choupana s/n, Santiago de Compostela 15706, Spain; General Surgery Department, Hospital Clinico Universitario de Santiago de Compostela, Rua Choupana s/n, Santiago de Compostela 15706, Spain; Pathology Department, Hospital Clinico Universitario de Santigao de Compostela, Rua Choupana s/n, Santiago de Compostela 15706, Spain; Plastic Surgery Department, Hospital Clinico Universitario de Santiago de Compostela, Rua Choupana s/n, Santiago de Compostela 15706, Spain

**Keywords:** pleomorphic lipoma, giant lipoma, lipoblasts

## Abstract

Pleomorphic lipomas are infrequent soft tissue tumours with pseudosarcomatous behaviour. Their polymorphism provides them certain characteristics that may resemble malignancy, which may mislead the initial diagnosis. The presented case report is a 45-year-old man with a giant growing tumour on the left cervicoscapular region initially categorised as a liposarcoma by magnetic resonance with a final confirmed diagnosis of pleomorphic lipoma after the surgical resection and the examination of the pathologist. The patient presented important functional restriction of the upper left extremity, which decreased after surgical resection, improving the quality of life.

## Introduction

Pleomorphic lipomas (PLs) are benign soft tissue tumours of adipose origin, clinically slow growing and generally asymptomatic, which appear more frequently on the shawl region of middle-aged men. Those acquiring a size larger than 10 cm or a weight heavier than 1000 g (<1% of PL) can be categorized as giant PL [1, 2]. Giant PL can cause symptoms such as pain or movement restrictions impairing the quality of life of the patient [1–3].

Definitive diagnosis is not always clear as PLs have a pseudosarcomatous behaviour that can mimic malignancy even though there is no risk of metastasis or malignant transformation. Radiologically, PLs lack pathognomonic diagnostic signs, resembling, on certain occasions, aggressive tumours and leading to an initial misdiagnosis. Clinical presentation and preoperative biopsies are crucial to elucidate the diagnosis of the patient and avoid radical surgery when not needed [4–6]. Pathological analyses showcases floret-like multinucleated giant cells (CD34+ and S100−), lipoblasts, and mature adipocytes (S100+) in a myxoid stroma with no signs of necrosis or mitotic activity suggesting malignancy [6–8]. Genetic molecular studies for MDMD2 and CDK4 in PLs are negative [2, 8, 9].

## Case report

**Figure 1:**
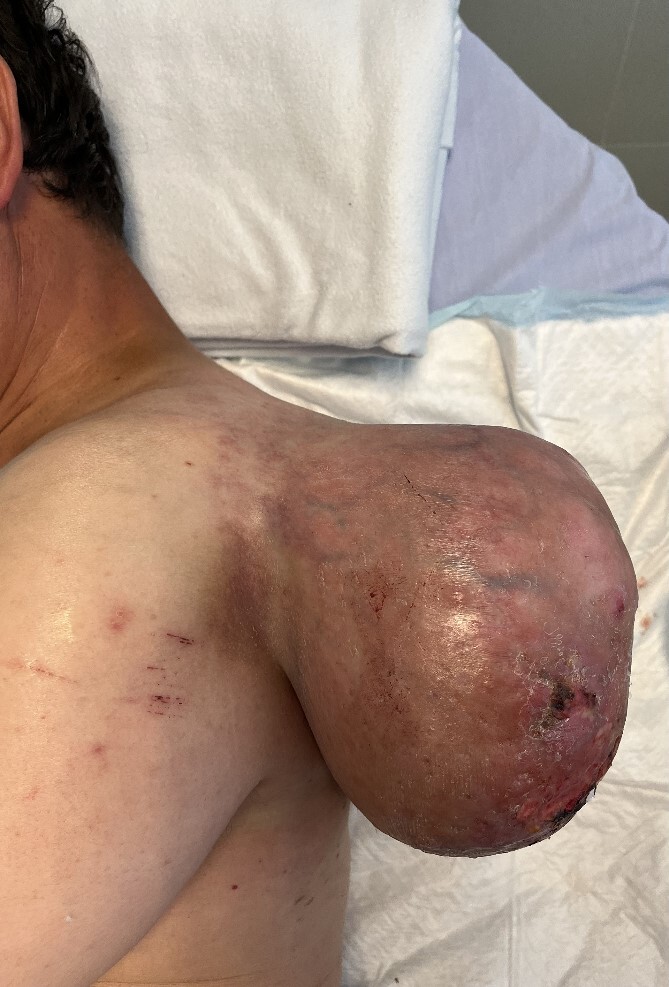
Giant soft tissue tumour on the left scapular region; initial signs of cutaneous pressure ulcers.

A 45-year-old man was admitted to the emergency department of our hospital as a result of a discontinuous bleeding in relation to a growing tumour located on his left shoulder (Fig. 1). The patient described a lesion of at least 20 years of evolution that started growing very rapidly over the past 12 months. Recently, the patient started noticing frequent blood spotting on his clothing, and for this reason, he attended the emergency department. In spite of the size of the lesion and functional restriction of the left upper limb, the patient declared himself capable of accomplishing his work obligations and daily activities without needing any help. The patient presented a previous diagnosis of osteochondromatosis and remained otherwise healthy.

Following a physical examination, the patient presented normal tensional values, no tachycardia, and no signs of paleness of the skin. An evident 20 cm mass attached to the cervicoscapular region of his left shoulder was found hanging from a pedicle ~10 cm in diameter. The lesion was dependent on the subcutaneous tissue and showed initial signs of cutaneous pressure ulcers. Signs of skin infection were found but no active bleeding was present. The lesion limited the patient’s mobility of the upper left extremity when trying to raise the shoulder with an extended arm; however, the remaining shoulder movements were preserved.

**Figure 2:**
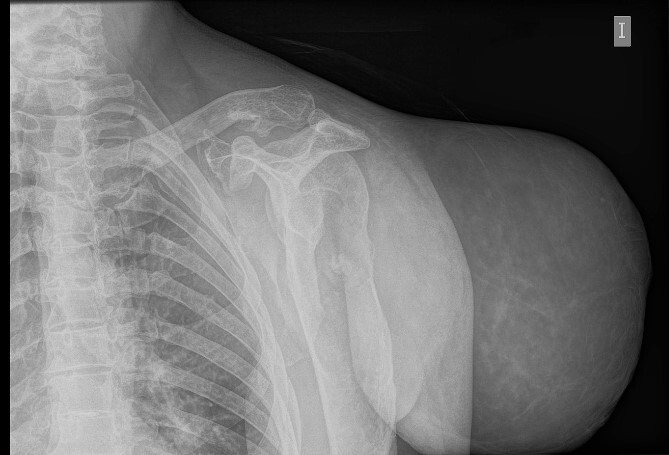
Simple radiography: evident soft tissue tumour on the left shoulder with multiple bone deformities compatible with osteochondromatosis.

An initial study was performed at the emergency department to dismiss extended disease in case of final proven malignancy. The laboratory results were within normal values; no anaemia was detected. Simple radiography described a giant soft tissue tumour and bone deformities compatible with osteochondromatosis (Fig. 2). The CT scan described a voluminous tumour of adipose tissue in the scapular region of the left shoulder together with other bone lesions compatible with multiple osteochondromatosis. No metastases were described in this study. The patient was discharged with oral antibiotics and the use of a shoulder sling to prevent further complications due to the weight of the tumour.

**Figure 3:**
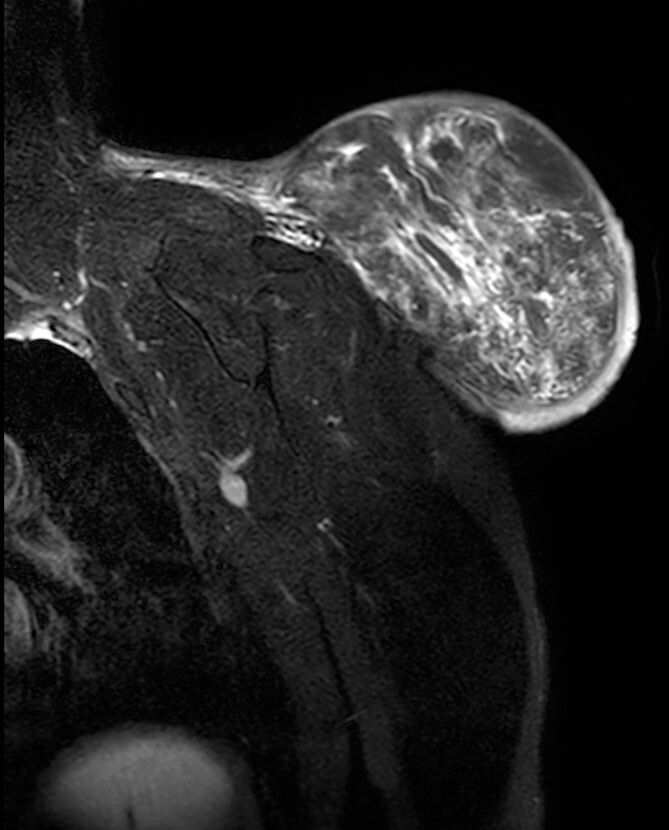
Magnetic resonance imaging T2 with contrast: giant excrescent lesion of 19 × 18 × 14.4 cm of diameter located in the subcutaneous tissue of the posterior aspect of the scapula divided by multiple septums; axillary lymph node of ~0.9 cm; images compatible with liposarcoma with a metastatic axillary lymph node.

**Figure 4:**
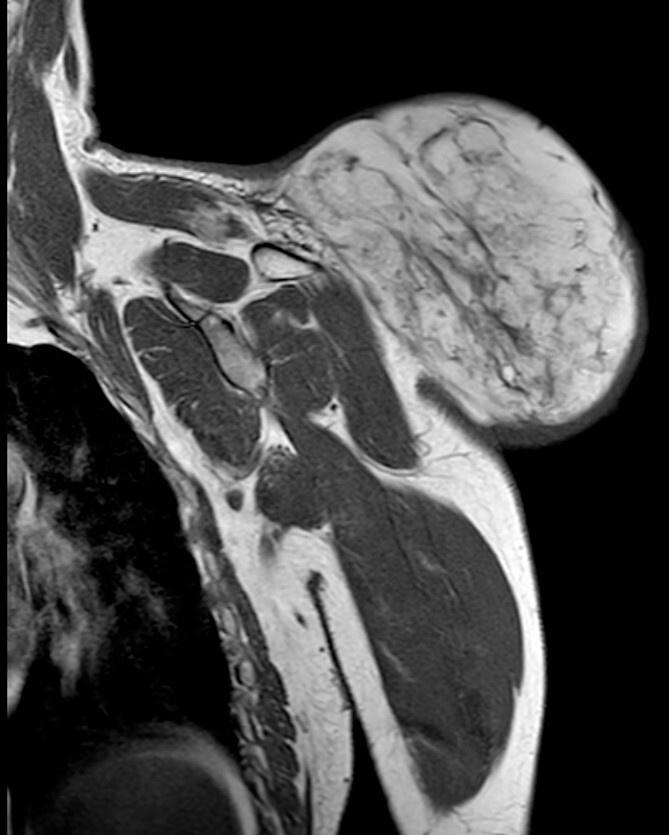
Magnetic resonance imaging T1: polylobulated lesion with multiple septa separating different areas of fat tissue.

During the follow-up, a magnetic resonance (Figs 3 and 4) was performed describing a soft tissue lesion highly suggestive for liposarcoma as a first possibility diagnosis, with an addition image suggesting metastatic axillary lymph node. After the imaging results, biopsies were taken and analysed by the pathologist with a final diagnosis of PL.

Immunohistochemistry studies revealed a diffuse and intense CD34 activity on the fusocelular and pleomorphic component and S100 immunoreactivity on the adipose component. CK AE1/AE3, EMA, AML, Desmin, SOX100, HMB45, CD31, STAT6, MDM2, and CDK4 were also tested but resulted negative. The FISH molecular study for the gene MDM2 was not amplified.

**Figure 5:**
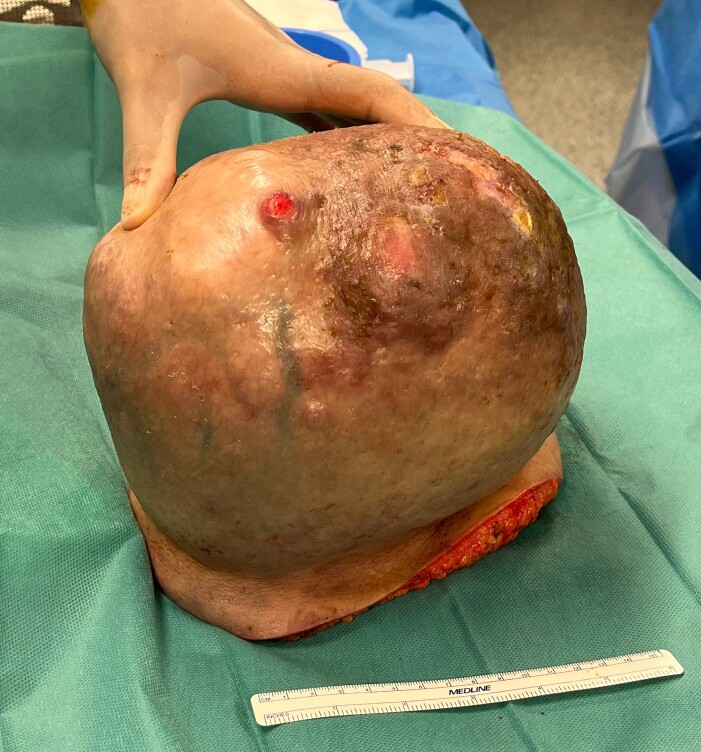
Macroscopic findings: solid lesion of 21 × 16 × 17cm of lipomatous characteristics with focus of fat necrosis compatible with PL.

**Figure 6:**
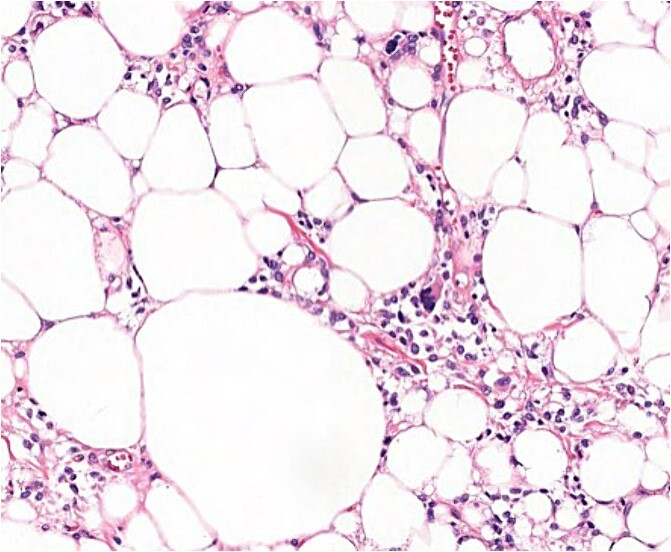
Microscopic findings (H & E ×40): well-circumscribed lesion at the profound dermis, composed of mature adipocytes, with occasional pleomorphic lipoblasts and spindle cells in the stroma some of which presented ‘floret’-like morphology; focal areas of necrosis and absence of mitotic activity; intense and diffuse response to CD34.

After the final diagnosis, surgery was scheduled in an ambulatory regime. Surgical excision of the tumour was performed generating a skin area defect on the left shoulder covered with a skin graft from the lateral aspect of the left thigh of the patient. The surgical piece presented lipomatous macroscopic characteristics with a total weight of 3110 g (Fig. 5). Under the microscope, floret-like spindle cells are presented, embedded together with mature adipocytes (Fig. 6).

**Figure 7:**
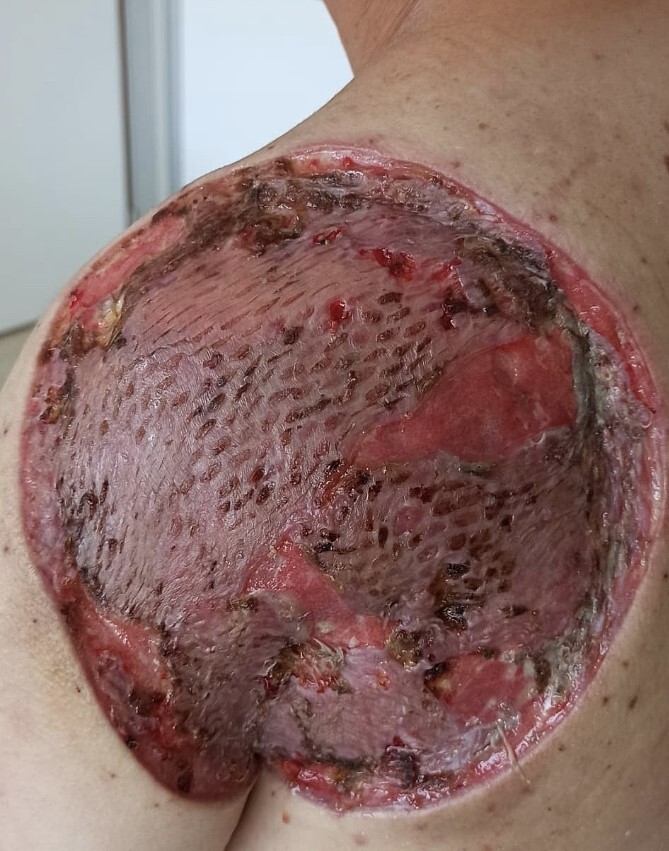
Skin graft after 3 week of the surgery; correct signs of healing, good vascularization, no necrosis.

The patient was discharged on the same day of the surgery with no immediate surgical complications. The skin graft remained with good vascularization and cicatrisation (Fig. 7). Healing was adequate, with some areas of skin hypertrophy that needed treatment with topic corticoids.

Rehabilitation therapy was started after the surgery to improve the range of movement of the shoulder; however after 1 year, the abduction achieved on the left shoulder was a maximum of 90 degrees. A Z - plasty-type surgery was performed to elongate the contracted scar and increase the patient’s movement capability.

## Discussion

PLs are a subtype of lipomas with a specific clinical presentation pattern and microscopic characteristics that generate an entity by themselves, different to other soft tissue tumours of adipose origin [8].

The most common location is the shoulder, the cervicoscapular region, and the upper back, appearing as a slow-growing, painless, mobile, and solitary lesion dependent of the subcutaneous tissue. These tumours are more frequent in men in comparison with women in a ratio of 9:1 and although they can occur at any age, they tend to appear around the fifth and seventh decade of the life. Size varies from 1.0 to 14.0 cm, being generally smaller than 5 cm [6, 8]. Sanchez *et al*. categorized giant lipomas as those bigger than 10 cm in at least one dimension or those heavier than 1000 g [1, 2], as is the case in this report with a total weight of more than 3000 g.

Giant lipomas are infrequent representing <1% of lipomas. Besides causing cosmetic and physical deformities, they affect the quality of life of the patient by causing compression, pain, and by diminishing their movement capacity [2, 3]. Recent reviews discard PL metastatic potential risk of malignant transformation [6]. However, Johnson *et al*. recommend that every soft tissue tumour larger than 5 cm should be managed as malignant until proven otherwise [10].

Soft tissue tumours from benign and malignant origin can mimic clinical presentation needing the microscopic analysis to help clarify the differential diagnosis [6]. Under the microscope, PLs are characterized for presenting typical floret-like multinucleated giant cells (CD34+ and S100−) embedded in a myxoid stroma with a variable distribution of mature adipocytes (S100+). No signs of infiltrative growth, necrosis, or mitotic activity are found [6–8]. The presence of lipoblasts is not uncommon in PLs, being present in around 66% of the cases; therefore, it is not considered a distinguishable clinical feature of liposarcoma [9, 11].

Typical immunochemistry results present negative desmin values, positive CD34, and negative S100 (positive S100 in the presence of mature adipocytes). Molecular genetic testing is considered the most reliable diagnostic tool [11], showing a lack of amplification of MDM2 or CDK4 in PLs, which helps differentiate them from other soft tissue tumours such as atypical lipomatous tumours or dedifferentiated liposarcomas (DDLS), which have positive nuclear expression for MDM2 or/and CDK4 in most cases [9, 12, 13].

To comprehend the radiographic behaviour of PLs is important; on certain occasions, superimposable images of malignant lesions can lead to the misdiagnosis of aggressive tumours [5, 9]. In contrast with an MRI, PL shows intense enhancement of the nonadipose component distinguishing PL from conventional lipomas; however, similar behaviour is found in soft-tissue sarcoma, which can lead to an erroneous diagnosis [5, 12, 13]. The adipose component is very variable in PLs (25%–75%). Low fat PLs can resemble more aggressive tumours like DDLS or non-adipocytic sarcomas [4, 6]. Vascularization analysis is also key, varying from a discreet vascularization to an abundant plexiform vascularization, which can simulate malignant tumours [4]. There are no pathognomonic signs for PLs on diagnostic imaging, which can delay the final diagnosis, the reason is clinical presentation and preoperative biopsies are vital to elucidate the diagnosis of the patient and avoid radical surgery when not needed [4, 5].

Surgery by simple excision with free margins is considered the gold standard with low rate of recurrence. Even though some studies declare better cosmetic outcomes and lesser morbidity when lipomas are treated by suction mechanisms, open surgery provides a smaller rate of complications, nerve damage, and a lower rate of recurrence [5, 14].

## Conclusions

PLs can emulate malignant soft tissue tumours in terms of clinical presentation, radiological imaging, and histopathological findings, leading from the daily clinical practice to an erroneous initial diagnosis. It is important to establish a multidisciplinary approach helped by an adequate imaging and pathology experts that may help to establish a correct final diagnosis and hence plan the type of surgical intervention. Free margins surgery provides good and safe oncologic results with low recurrency rates.
